# Three curved thoracic left-scoliosis after tethered spinal cord surgery

**DOI:** 10.1186/1748-7161-8-S2-O13

**Published:** 2013-09-18

**Authors:** Petra Gröbl, Christa Lehnert-Schroth

**Affiliations:** 1 Degree Program Physiotherapy, FH Joanneum Graz, Austria

## Background

The case study describes a patient with scoliosis after tethered spinal cord-symptomatic was surgically resolved at the age of 2½ months. At that time, the Cobb angle of the thoracic curve was 32°. At the age of 30 months a three curved thoracic left-scoliosis with a Cobb angle of 80° with serious shape-shift in frontal and sagittal level appeared. At the age of 4½ years the patient was fitted with a Rigo-Cheneau corset, which guided the breath into the right side.

## Methods

At the age of 36 months, the Schroth treatment was started. At this time, the primary goal was the correction of the hip that was positioned outwards to the right. This sideward movement, combined with a twist of the pelvis forced the thoracic cage into convexity on the left side and left-backward rotation, producing rib prominence. A hyper-correction according to Schroth was instructed: Side-lying on the right, a thick padding underneath the right hip, flexion with lengthening force to cranial with the right arm. The concave side was wide-positioned, thus diverting the breath in that direction. The right ribs showed two tensed areas. Schroth’s turn-angle-breathing was always asked for at the concave places and carried out three-dimensionally in connection with the considered lowering of the diaphragm, eventually repositioning the “weak” ribs into their normal place.

## Results

At the beginning of the treatment, there were two visible tensed rib areas, which required tactile stimulus as well as expanse and breathing. After a few months, the right side became stretched and appeared nearly normal. At 2½ years: Body limp and weak, X ray 80° Cobb. At 5½ years: Posture upright, pelvis straight, body appears steady, incorrect posture of the head equable, X ray 45° Cobb.

## Conclusions and discussion

The Schroth method seems to be significantly effective even in scoliosis with contributing neurogenic factors.
